# A case of retrograde intussusception at Roux-en-Y anastomosis 10 years after total gastrectomy: review of the literature

**DOI:** 10.1186/s40792-016-0250-6

**Published:** 2016-11-03

**Authors:** Yuhei Kitasato, Ryuta Midorikawa, Yoshihiro Uchino, Shuko Saku, Taizan Minami, Takahisa Shirahama, Kazumitsu Kiyomatsu, Koji Okuda, Yoshito Akagi, Hiroyuki Tanaka

**Affiliations:** 1 Department of Surgery, JCHO Saga Central Hospital, 3-8-1 Hyogo-minami, Saga, Saga 849-8522 Japan; 2 Department of Surgery, Kurume University School of Medicine, 67 Asahi-machi, Kurume, Fukuoka, 830-0011 Japan

**Keywords:** Total gastrectomy, Retrograde intussusception, Roux-en-Y anastomosis

## Abstract

A 63-year-old man, who had undergone total gastrectomy and Roux-en-Y reconstruction for gastric cancer 10 years previously, was admitted to our hospital with complaints of abdominal pain, palpable abdominal tumor, and hematemesis. On admission, the abdominal tenderness was improving and no abdominal tumor was palpable. Mild inflammatory changes and anemia were noted on blood examination. Abdominal computed tomography revealed a tumor with a layered structure in the left abdomen. The patient was diagnosed with intestinal obstruction secondary to intussusception, and surgery was performed. Retrograde intussusception was found at the site of the Y anastomosis. We conducted manual reduction using the Hutchinson procedure. The intestinal color after the reduction was good, and no intestinal resection was required. Postoperative recovery was uneventful, and the patient was discharged 12 days after surgery. Reports of jejunal intussusception after total gastrectomy with Roux-en-Y reconstruction are relatively rare. Here, we report a case of jejunal intussusception after total gastrectomy with Roux-en-Y reconstruction.

## Background

Intussusception occurs when a portion of intestine invaginates into an adjacent section of intestine. Common physical exam findings in adults with intussusception include intermittent abdominal pain, vomiting, gastrointestinal bleeding, and/or the presence of a palpable mass. Possible sequelae of intussusception include small bowel obstruction and ischemia. Jejunal intussusception is a rare complication after gastrectomy and extremely rare after total gastrectomy. In this case study, we report a case of retrograde intussusception at Roux-en-Y anastomosis occurring 10 years after total gastrectomy. We also review other cases of intussusception after total gastrectomy that are reported in the literature.

## Case presentation

A 63-year-old man, who had undergone total gastrectomy and Roux-en-Y reconstruction for early gastric cancer 10 years previously, was transferred to our institution from a local hospital with complaints of intermittent abdominal pain, palpable abdominal tumor, and hematemesis. On arrival, he was hemodynamically stable, the abdominal tenderness was improving, and the abdominal tumor was not felt on physical examination. Mild inflammatory reaction and anemia were noted on blood examination. All other laboratory values were unremarkable and there was no evidence of acidosis.

**Fig. 1:**
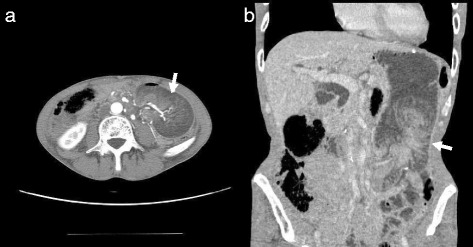
**a**, **b** Abdominal computed tomography showing a tumor with a layered structure in the left abdomen (*white arrow*). Intestinal obstruction secondary to intussusception was suspected

**Fig. 2:**
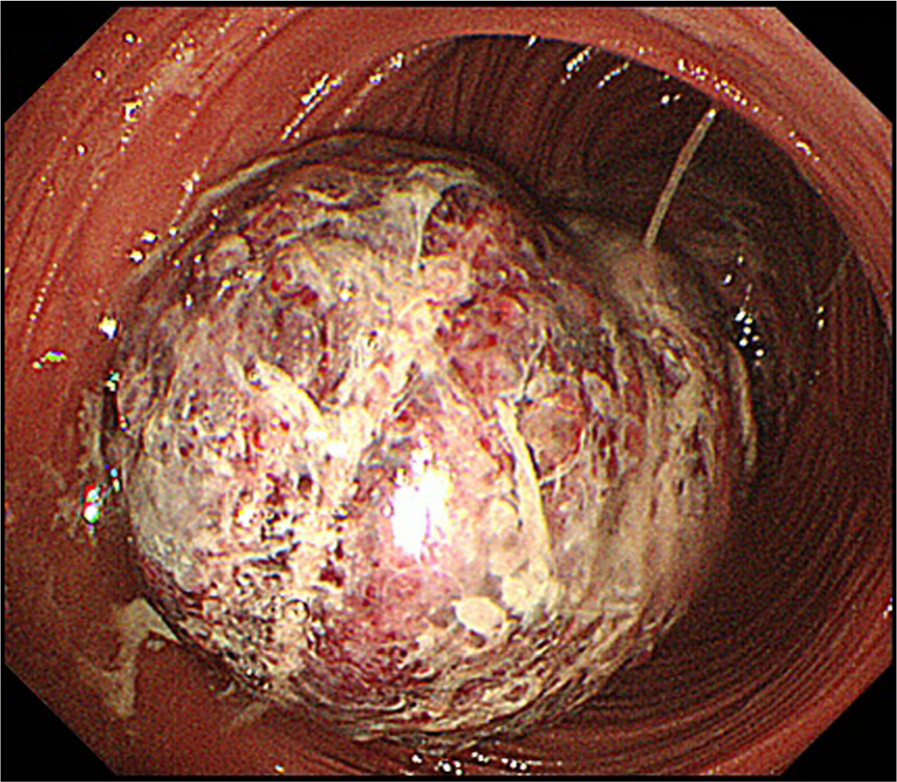
Upper gastrointestinal endoscopy revealed congested mucosa of the small intestinal intussusception

Abdominal computed tomography (CT) revealed a tumor with a layered structure in the left abdomen (Fig. 1). Endoscopy revealed a bulky, rounded, congested mass that occupied the lumen beyond the esophagojejunal anastomosis (Fig. 2) and showed a small amount of oozing from the mucosal surface caused by disruption to the blood flow resulting from intussusception. These findings led to the diagnosis of intestinal obstruction caused by jejunal intussusception. Although we observed retrograde jejunal intussusception, endoscopic reduction was not attempted and surgical repair was performed by laparotomy.

**Fig. 3:**
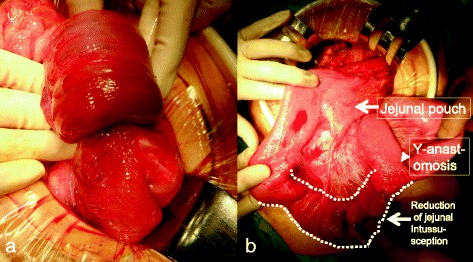
**a**, **b** Intraoperatively, a retrograde intussusception at the Y-anastomosis was observed (**a**). Manual reduction was performed using the Hutchinson procedure (**b**). The color of intussuscepted intestine after the reduction was good, and intestinal resection was unnecessary (*black arrow*: jejunal pouch, *white arrowhead*: Y-anastomosis, surrounded by the *dotted line* and *white arrow*: intussuscepted intestine)

Surgery revealed retrograde jejunal intussusception in the elevated jejunum through the Y anastomosis (Fig. 3a). We reduced the intussusception using the Hutchinson procedure. The involved bowel was dilated, edematous, and congested, but there was no evidence of ischemia (Fig. 3b), and therefore, we performed only manual reduction and adhesiotomy without intestinal resection. The patient’s postoperative recovery was free of complications, and he was discharged 12 days postoperatively. He has been alive without symptoms of bowel obstruction for 2 years after operation.

## Discussion

Jejunal intussusception after gastrectomy was first reported by Bozzi [1] and is recognized as an uncommon complication, occurring in only 0.07–2.1% of patients who undergo gastrectomy [2]. Intussusception is attributed to both mechanical factors (excessive length of afferent loop, lifting afferent loop to the stomach wall, excessively large anastomosis hole, ptosis of the gastrojejunal anastomosis, postoperative adhesions, and stenosis causing reverse peristalsis) and functional factors (spasm of the intestine, peristaltic abnormality, surgical stimulation, inflammation, autonomic nervous system abnormality, enteral nutrition, drug infusion, and decrease in gastric wall tension) [2, 3]. At the site of the Roux-en-Y anastomosis in this case, adhesions were mild and no strictures or mass were observed that would cause reverse peristalsis. However, mechanical factors cannot be entirely ruled out in this patient; we suspect that some kind of peristaltic abnormality may have occurred. Tu and Kelly reported reverse peristalsis caused by an apparent ectopic pacemaker in a Roux-en-Y anastomosis of small intestinal resection [4]. This clinical condition may also cause retrograde intussusception after total gastrectomy and Roux-en-Y reconstruction.

Jejunal intussusception after total gastrectomy is rare. A review of the literature revealed 18 cases of intussusception occurring after total gastrectomy with Roux-en-Y reconstruction, including the current case (Table 1) [3, 5–18]. The majority of patients experiencing this complication were 60–70 years old. Only four cases of antegrade intussusception were observed; the other cases were retrograde intussusception. Furthermore, only six cases developed in the early postoperative period; other cases developed 1–22 years after surgery.

**Table 1:** Cases of jejunal intussusception after total gastrectomy with Roux-en-Y reconstruction

Case	Age	Sex	Diagnosis	Time after gastrectomy	Type of intussusception	Treatment	Year of reported	Author
1	63	M	Gastric cancer	3 years	Retrograde	None	1954	Davey [3]
2	48	F	Gastric cancer	23 days	Retrograde	Partial resection of jejunum	1965	Nishi
3	65	M	Gastric cancer	6 days	Retrograde	Manual reduction	1965	Kato et al. [5]
4	40	M	Sarcoma	5 years	Retrograde	Partial resection of jejunum	1966	Freeman et al.[6]
5	39	F	Gastric cancer	16 days	Antegrade	Manual reduction	1984	Hanyu et al. [7]
6	61	F	Gastric cancer	12 years	Retrograde	Manual reduction	1993	Hashimoto et al. [8]
7	58	F	Gastric cancer	1 year	Retrograde	Partial resection of jejunum	1994	Narushima et al. [9]
8	75	M	Esophageal cancer	9 years	Retrograde	Manual reduction	2000	Goto et al. [10]
9	50	F	Gastric cancer	10 days	Antegrade	Manual reduction	2001	Ozdogan et al. [11]
10	60	M	Gastric cancer	4 years	Retrograde	Manual reduction	2005	Akiyama et al. [12]
11	74	M	Gastric cancer	12 years	Retrograde	Partial resection of jejunum	2005	Matsumoto et al. [13]
12	74	M	Gastric cancer	21 years	Retrograde	Manual reduction	2006	Sato et al. [14]
13	75	M	Gastric cancer	10 years	Antegrade	Manual reduction	2010	Ueno et al. [15]
14	69	M	Gastric cancer	45 days	Antegrade	Manual reduction	2012	Matsuda et al. [16]
15	77	F	Gastric cancer	5 days	Retrograde	Manual reduction	2013	Lee et al. [17]
16	75	M	Gastric cancer	21 years	Retrograde	Manual reduction	2013	Kita et al. [18]
17	76	M	Gastric cancer	22 years	Retrograde	Partial resection of jejunum	2013	Kita et al. [18]
18	63	M	Gastric cancer	10 years	Retrograde	Manual reduction	2015	Our case

In 12 of the 18 reported cases, enterectomy was not performed. Kita et al. reported recurrence of intussusception within 1 year of manual reduction of intussusception. Recurrence may be more likely when only manual reduction is used, and therefore, resection and re-anastomosis should be considered [18]. We think that we may prevent retrograde intussusception by making Y leg side-to-side anastomosis in the case of gastrectomy. This is because it thinks that it may do intussusception by the peristalsis that was handed down to intestinal tract by making end-to-side anastomosis.

## Conclusions

We report a case of retrograde intussusception at Roux-en-Y anastomosis 10 years after total gastrectomy.

## Abbreviations

CT: Computed tomography
